# Supplementary material for: Hybrid Models and Biological Model Reduction with PyDSTool
Source: PLoS Comput Biol. 2012 Aug 9;8(8):e1002628. doi: 10.1371/journal.pcbi.1002628 (PMC3415397; doi:10.1371/journal.pcbi.1002628)
Supplement: Text S4 — Complete source code for the PyDSTool package (version 0.88.120504). Includes API documentation and help files linking to web pages. This file is identical to the current public release on Sourceforge.net. (ZIP) [file pcbi.1002628.s004.zip › PyDSTool/html/PyDSTool.Toolbox.neuro_data.burst_feature-class.html]

xml version="1.0" encoding="ascii"?


PyDSTool.Toolbox.neuro\_data.burst\_feature


| Home | Trees | Indices | Help | PyDSTool |
| --- | --- | --- | --- | --- |

Package PyDSTool ::
Package Toolbox ::
Module neuro\_data ::
Class burst\_feature

[hide private]

[frames] | no frames]

# Class burst\_feature

source code

```
       object --+            
                |            
 MProject.feature --+        
                    |        
MProject.feature_node --+    
                        |    
 MProject.ql_feature_node --+
                            |
                           burst_feature
```

---

Embed the following sub-features, if desired: get\_burst\_X, where X is
a number of feature types defined in this module.


|  |  |
| --- | --- |
| Instance Methods | [hide private] |

|  |  |
| --- | --- |
| \_local\_init(self) | source code |

|  |  |
| --- | --- |
| postprocess\_ref\_traj(self)  User-definable by overriding in a sub-class | source code |

|  |  |
| --- | --- |
| prepare(self, target)  Operations to prepare for testing (optional). | source code |

**Inherited from `MProject.feature_node`**:
`__getitem__`,
`__init__`,
`__repr__`,
`__str__`,
`evaluate`,
`propagate_verbosity`,
`set_ref_traj`

**Inherited from `MProject.feature_node`** (private):
`_residual_info`

**Inherited from `MProject.feature`**:
`__call__`,
`__eq__`,
`__hash__`,
`__ne__`,
`finish`,
`info`,
`reset_metric`,
`validate`

**Inherited from `MProject.feature`** (private):
`_find_idx`

**Inherited from `object`**:
`__delattr__`,
`__getattribute__`,
`__new__`,
`__reduce__`,
`__reduce_ex__`,
`__setattr__`


|  |  |
| --- | --- |
| Properties | [hide private] |

**Inherited from `object`**:
`__class__`


|  |  |
| --- | --- |
| Method Details | [hide private] |

|  |  |
| --- | --- |
| postprocess\_ref\_traj(self) | source code |

User-definable by overriding in a sub-class

Overrides: MProject.feature.postprocess\_ref\_traj: *(inherited documentation)*

|  |  |
| --- | --- |
| prepare(self, target) | source code |

Operations to prepare for testing (optional). Override in concrete
sub-class if desired

Overrides: MProject.feature.prepare: *(inherited documentation)*

  


| Home | Trees | Indices | Help | PyDSTool |
| --- | --- | --- | --- | --- |

|  |  |
| --- | --- |
| Generated by Epydoc 3.0.1 on Fri May 4 15:24:09 2012 | http://epydoc.sourceforge.net |
